# Advances in Ultrasound-Guided Surgery and Artificial Intelligence Applications in Musculoskeletal Diseases

**DOI:** 10.3390/diagnostics14182008

**Published:** 2024-09-11

**Authors:** Soichi Hattori, Rachit Saggar, Eva Heidinger, Andrew Qi, Joseph Mullen, Brianna Fee, Cortez L. Brown, Stephen P. Canton, Devon Scott, MaCalus V. Hogan

**Affiliations:** Foot and Ankle Injury Research (FAIR), Division of Foot and Ankle, Department of Orthopaedic Surgery, University of Pittsburgh Medical Center, Pittsburgh, PA 15219, USA; saggarrachit15@gmail.com (R.S.); heidingerem@upmc.edu (E.H.); andychi62@gmail.com (A.Q.); jpm399@georgetown.edu (J.M.); feebk0@sewanee.edu (B.F.); brownc68@upmc.edu (C.L.B.); cantonsp@upmc.edu (S.P.C.); scottd18@upmc.edu (D.S.); hoganmv@upmc.edu (M.V.H.)

**Keywords:** musculoskeletal ultrasound, ultrasound-guided surgery, artificial intelligence, deep learning, radiomics

## Abstract

Ultrasound imaging is a vital imaging tool in musculoskeletal medicine, with the number of publications on ultrasound-guided surgery increasing in recent years, especially in minimally invasive procedures of sports, foot and ankle, and hand surgery. However, ultrasound imaging has drawbacks, such as operator dependency and image obscurity. Artificial intelligence (AI) and deep learning (DL), a subset of AI, can address these issues. AI/DL can enhance screening practices for hip dysplasia and osteochondritis dissecans (OCD) of the humeral capitellum, improve diagnostic accuracy for carpal tunnel syndrome (CTS), and provide physicians with better prognostic prediction tools for patients with knee osteoarthritis. Building on these advancements, DL methods, including segmentation, detection, and localization of target tissues and medical instruments, also have the potential to allow physicians and surgeons to perform ultrasound-guided procedures more accurately and efficiently. This review summarizes recent advances in ultrasound-guided procedures for musculoskeletal diseases and provides a comprehensive overview of the utilization of AI/DL in ultrasound for musculoskeletal medicine, particularly focusing on ultrasound-guided surgery.

## 1. Introduction

Musculoskeletal (MSK) ultrasound is an indispensable imaging modality, first developed in diagnostics [1,2,3] and now in the management of various MSK disorders [4,5]. Its ability to provide real-time, dynamic assessment of musculoskeletal structures makes it particularly valuable in clinical practice. MSK ultrasound offers numerous advantages, including high spatial resolution, absence of ionizing radiation, and the ability to perform bedside examinations, thus facilitating prompt clinical decision making. Additionally, its cost-effectiveness compared to other imaging modalities, such as MRI, has further cemented its role in the routine evaluation of MSK conditions. The versatility of MSK ultrasound enables clinicians to visualize soft tissue structures, such as muscles, tendons, ligaments, and nerves, as well as to guide therapeutic procedures with precision and safety [6].

However, despite these advantages, MSK ultrasound is not without its limitations. The modality is highly operator-dependent, requiring significant expertise to acquire and interpret images accurately. This dependency can lead to variability in diagnostic accuracy and procedural outcomes. Furthermore, the quality of the images can be compromised by factors such as patient body habitus, presence of soft tissue artifacts, and the inherent limitations of the ultrasound technology itself [7]. These challenges underscore the need for advanced techniques and tools to enhance the reliability and utility of MSK ultrasound in clinical practice.

Artificial intelligence (AI) encompasses several branches of data science that specialize in various domains. Deep learning (DL), a subset of machine learning which itself is a subset of AI, consists of frameworks of neural networks that accomplish data processing and are designed to mimic human cognitive abilities [8]. It depends on the availability of large amounts of data and algorithms to be efficient. Specifically, two-dimensional and three-dimensional convoluted neural networks (CNNs) along with other architectures can detect and analyze visual features with a high degree of accuracy that is often faster and more efficient than traditional methods. Furthermore, other forms of AI and semi-supervised learning can provide proofreading capabilities for image analyses performed by DL [6].

Ultrasound imaging is not viewed from standardized planes like other medical imaging technology, making it vulnerable to unclear graphics that are difficult and time-consuming to interpret manually [6]. As DL has gained traction in the past two decades as a useful tool, its application in clinical settings and MSK medicine has become important to remedy the disadvantages of ultrasound imaging mentioned previously. It has proven to be helpful for anatomical segmentation, localization and removal, release and cutting, and the repair of targeted afflictions [5]. The integration of AI/DL into MSK ultrasound represents a significant advancement in medical imaging.

This review aims to address the following key questions: (1) How extensively has ultrasound-guided surgery been explored in MSK medicine and orthopedics? (2) In what ways are AI and DL technologies being utilized to enhance the diagnostic capabilities and outcomes in MSK ultrasound-guided surgeries? By investigating these questions, the paper seeks to provide a comprehensive overview of the current state of research and the potential future impact of AI/DL on ultrasound-guided procedures in MSK medicine.

## 2. Ultrasound-Guided Surgery for Musculoskeletal Diseases

### 2.1. Literature Search

**Figure 1:**
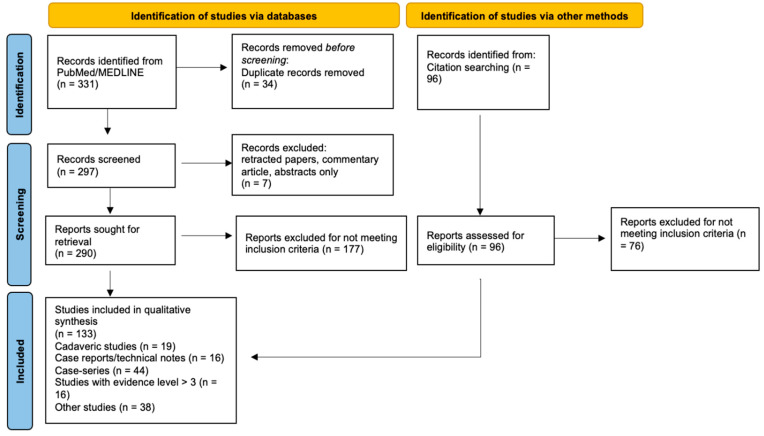
Flowchart depicting the literature search methodology for ultrasound-guided surgery in musculoskeletal disease.

In the present review of ultrasound-guided surgery, we used the PubMed database to conduct a comprehensive literature search covering studies published from inception to June 2024. The search algorithm included combinations of the following keywords: “ultrasound-guided”, “sonographically-guided”, “ultrasonography-guided”, “ultrasound-assisted”, “sonographically-assisted”, “ultrasonography-assisted”, or “intraoperative ultrasound”. These terms were paired with “orthopedic”, “musculoskeletal”, “ligament”, “tendon”, or “nerve”. We excluded studies related to “biopsy”, “block”, “injection”, “anesthesia”, “pain”, “electromyography”, “catheter”, “aspiration”, “dry needling”, “radiofrequency ablation”, “electrolysis”, or “hydrodistension”, as well as those focused on pre-operative evaluation by ultrasound, to maintain a focus on intraoperative applications. Boolean operators (AND, OR) were used to combine search terms appropriately. Additionally, to ensure comprehensive coverage, a snowball approach was also conducted, manually searching references from relevant articles (Figure 1).

### 2.2. Classification of Studies

After removing duplicates, retracted papers, and narrative reviews, two independent reviewers screened titles and abstracts, followed by full-text review of potentially eligible studies. Disagreements were resolved through discussion or by consulting a third reviewer.

A total of 133 studies met the inclusion criteria for the literature search. Data extracted from these studies included study design (e.g., cadaveric studies, case report/technical note, case series, comparative studies, randomized control trial, and meta-analysis/systematic review), procedure type performed (e.g., localization, release, repair, etc.), pathology (e.g., carpal tunnel syndrome, Achilles tendon rupture, plantar fasciitis, etc.), targeted tissues (e.g., nerve, tendon, fascia, etc.), sample size, and key findings of each study. 

### 2.3. Definition of Ultrasound-Guided Surgery 

It is important to note the difference between ultrasound-guided and ultrasound-aided/assisted surgery. Ultrasound-guided surgeries included those where the entire surgical procedure was performed with ultrasound guidance. Examples included ultrasound-guided carpal tunnel release and trigger finger release. Ultrasound-assisted surgeries included those where ultrasound was used for specific parts of a surgical procedure, for instance, using ultrasound to assist in identifying portal entry sites for arthroscopic surgery. In Achilles tendon repair, the procedure was classified as ultrasound-assisted surgery when intraoperative ultrasound was used solely to identify the course of the sural nerve [9]. It was considered ultrasound-guided surgery when ultrasound was utilized to detect the sural nerve as well as the sutures and/or needle within the Achilles tendon during the repair [10]. 

### 2.4. Ultrasound-Assisted Surgery

Ultrasound-assisted surgery composed 38 out of the 133 studies reviewed. The ultrasound capacity to accurately detect targeted anatomical/pathological structures was utilized for a portion of the surgical procedures. During arthroscopic/endoscopic and even trauma surgery, ultrasound was used to accurately identify a joint space or critical landmarks and/or nerves and arteries for accuracy and safety [11,12,13,14,15,16,17]. Tumors, small ossicles, and calcifications, which were not possible to detect with conventional C-arm X-ray, could be localized with intraoperative ultrasound to facilitate open or arthroscopic removal [18,19,20]. Of note, ultrasound was used to localize and/or confirm decompression during spine surgery with a systematic review that validated its efficacy [21]. 

### 2.5. Classification of Ultrasound-Guided Surgery

The procedure types of ultrasound-guided surgery can be classified into 3 categories: 1. localization and removal/debridement, 2. release or cutting (partial or complete), 3. repair. Ultrasound allows for accurately localizing and removing (completely or partially) pathological tissues, and these procedures are referred to as the “first generation” of ultrasound-guided surgery [5]. Release or cutting of targeted structures including tendon, fascia, retinaculum, etc. is known as the “second generation” while ultrasound-guided repairs are called the “third generation” [22,23,24,25]. 

The review of 95 studies on ultrasound-guided surgery revealed that five “first generation” surgeries involved removing/debriding foreign bodies [26,27], hematoma [28,29], and excessive bone and bursa in Haglund deformity [30,31,32].

The second generation procedures were most common and involved release/cutting of soft tissues, including transverse carpal ligament release for carpal tunnel syndrome [1,33,34,35,36,37,38,39,40,41,42,43,44,45,46], flexor retinaculum release and septum for tarsal tunnel syndrome [47,48,49,50], shoulder capsule/coracohumeral ligament for adhesive capsulitis [51,52,53,54], cutting of gastrocnemius aponeurosis to lengthen Achilles tendon [55,56,57], tendon sheath release for trigger fingers and DeQuervain tenosynovitis [58,59,60,61,62,63,64,65], fasciotomy (complete cutting) for chronic exertional compartment syndrome [66,67,68], fasciotomy (partial cutting) for Dupuytren contracture [69,70,71], tenotomy (complete cutting) of long head biceps and plantaris tendon for shoulder pain and Achilles tendinopathy, respectively [72,73,74,75,76,77,78,79,80,81,82,83,84,85,86], and partial tenotomy/fasciotomy including Tenex^®^ Lake forest, CA, USA for tendinopathy/fasciopathy [87,88,89,90,91,92,93,94,95]. 

Ultrasound-guided repairs are the “third generation” due to the novelty of their techniques. They comprised ultrasound-guided anterior talofibular ligament of the ankle [22,96,97], Achilles tendon [10,23,24,25,98,99], and medial collateral ligament and medial patellofemoral ligament of the knee [100,101].

### 2.6. Cadaveric Studies, Case Reports/Technical Notes, and Case Series

Among ultrasound-guided surgery, 19 cadaveric studies, 16 case reports/technical notes, and 44 case series were included. There were overlaps between the procedures in cadaveric studies and case series, indicating the advent and evolution of these techniques. Researchers initially conducted cadaveric studies to verify accuracy and feasibility, then progressed to a case series of their ultrasound-guided procedures on patients.

The second generation ultrasound-guided surgery, release or cutting of targeted structures, composed 86% of the 44 case series (the specific procedures are not clear in one case series), followed by localization and removal/debridement (the first generation) at 7% and repair (the third generation) at 7%.

### 2.7. Clinical Studies above Evidence Level 3

In 16 studies with an evidence level higher than 3 [102], targeted tissues of ultrasound-guided surgery included nerves, tendons, bursae, bone, and ligaments (Table 1). All of them were not evaluated or hard to identify with intraoperative C-arm X-ray.

Tendinopathy surgery was most common (*N* = 6) [84,93,103,104,105,106], followed by foot and ankle surgery (*N* = 5) [10,31,92,97,99] and hand surgery (*N* = 4) [44,45,46,64], among studies with higher evidence.

### 2.8. Randomized Control Trials and Meta-Analysis

The randomized controlled trials (RCTs) and meta-analysis on tendinopathy and fasciopathy suggested that US-guided procedures resulted in minimal complications. Across the studies, pain scores typically decreased significantly in the ultrasound-guided groups. Functional improvements were noted for ultrasound-guided procedures. General well-being, sleep quality, and function all showed positive trends. However, most of these results were not significant between ultrasound-guided and control groups. 

The RCTs on carpal tunnel syndrome and trigger finger release collectively indicate that ultrasound-guided procedures exhibit favorable safety with no significant complications compared to control groups. Pain levels were significantly lower in the ultrasound-guided group [45]. Functional improvement was noted in every study, although the functional improvements were not significant between the ultrasound-guided and control groups in some studies [46,64]. Notably, ultrasound guidance led to earlier functional recovery in one study [45] and demonstrated a quicker return to normal activities and better cosmetic outcomes in the other study [64].

Another RCT on shoulder capsule/coracohumeral ligament release for adhesive capsulitis found that ultrasound-guided coracohumeral ligament release with Tenex^®^ improved shoulder range of motion, pain, and function compared with local anesthetic injection group [54]. 

### 2.9. Strength of Ultrasound-Guided Surgery for Musculoskeletal Pathologies

**Figure 2:**
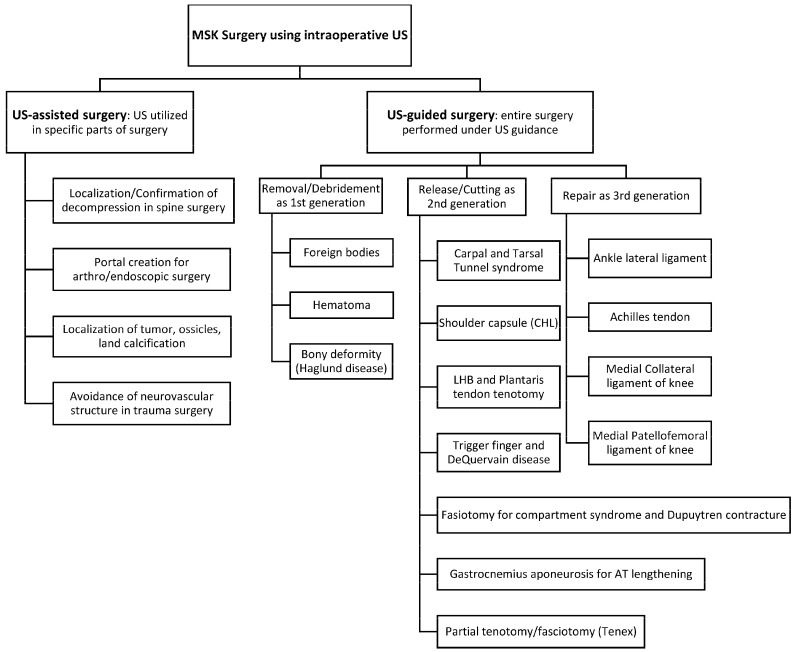
Summary of Ultrasound-guided and -assisted surgery for targeted pathologies. MSK, musculoskeletal; US, ultrasound; CHL, coracohumeral ligament; LHB, long head of biceps; AT, Achilles tendon.

As shown in the studies above, ultrasound-guided procedures demonstrated a strong safety profile and efficacy comparable to traditional methods, offering benefits in pain reduction and expeditious functional improvements. Intraoperative ultrasound can assist physicians and surgeons in accurately and effectively performing minimally invasive surgeries for soft tissue pathologies, particularly those not visible with intraoperative fluoroscopy (Figure 2).

## 3. Artificial Intelligence and Musculoskeletal Ultrasound 

### 3.1. Literature Search 

**Figure 3:**
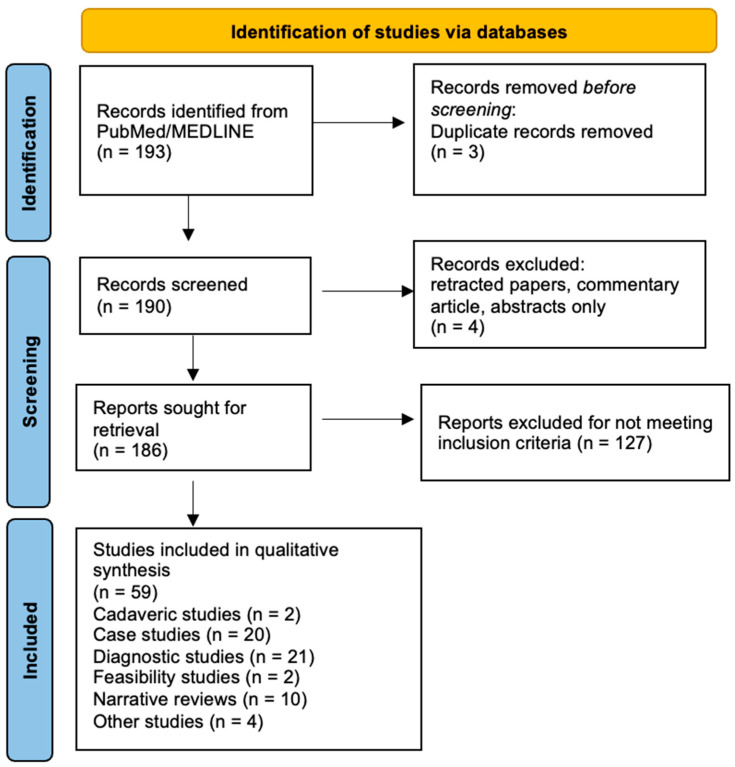
Flowchart depicting the literature search methodology for artificial intelligence and musculoskeletal ultrasound.

We searched the PubMed database from inception to June 2024 using a search strategy including combinations of the following keywords: “ultrasound-guided”, “ultrasonography-guided”, “ultrasound-assisted”, “sonography-assisted”, “ultrasonography-assisted”. These terms were paired with “orthopedic” and “musculoskeletal”, in combination with “deep learning”, “artificial intelligence”, “convolutional neural networks”, and “machine learning”. To focus on studies with diagnostic or screening potential, “diagnosis” and “screening” were added where relevant. The asterisk (*) symbol was used to include all variations of the above words. Boolean operators (AND, OR) were used to combine search terms appropriately (Figure 3). 

### 3.2. Classification of Studies 

After removing duplicates, retracted papers, and narrative reviews, two independent reviewers screened titles and abstracts, followed by full-text review of potentially eligible studies. Disagreements were resolved through discussion or by consulting a third reviewer. 

A total of 59 studies were included for the investigation of how AI and DL are used with ultrasound in MSK medicine and orthopedics. From these studies the following data were extracted: type of imaging used (e.g., ultrasound, MRI, X-ray), the role of AI in the study (e.g., detection, segmentation, classification, etc.), the state of the images or subjects used (e.g., abnormal, healthy), investigated anatomy or pathology (e.g., tendinopathy, hip dysplasia, carpal tunnel syndrome), reference standard (if any), type of study (e.g., narrative review, diagnostic, cadaveric), level of evidence (if any), and key findings of each study. 

### 3.3. Inclusion Criteria and Definitions of Artificial Intelligence, Deep Learning, and Convolutional Neural Network 

The inclusion criteria for these studies necessitated that the computer model conformed to the established definitions of artificial intelligence (AI), deep learning (DL), or convolutional neural networks (CNNs). AI was defined as machines that are programmed to think and learn in a simulation of human intelligence. It encompasses a variety of technologies and applications, including machine learning, natural language processing, and robotics. DL was defined as a subset of machine learning that involves neural networks with many layers that are capable of automatically extracting and learning features from data. CNNs were defined as a type of DL algorithm specifically designed for processing structured grid data, like images that consist of multiple layers, that applies convolutional operations to learn features from input images. 

Other inclusion criteria were developed based on these definitions and required the role of a computer in the study to detect an anatomical area or pathology from ultrasound, classify and/or diagnose a detected anatomical area or pathology from ultrasound, enhance ultrasound images for interpretation, or segment ultrasound images. 

### 3.4. Types of Studies 

Between 2017 and 2020, no diagnostic studies were performed, and most studies focused on developing algorithms and utilizing AI/DL/CNN for segmenting and tracking ultrasound images. The subjects of these studies were mostly healthy and/or cadaver models. Of the 59 studies included, 2 were categorized as cadaveric, 2 as feasibility-based, 10 as narrative reviews, and 20 as miscellaneous case studies, most of which were looking at segmentation and tracking. 

From 2021, however, the number of clinical studies increased, and 21 of the 58 studies were categorized as a “diagnostic study” and given an evidence level based on the guideline from *Journal of Bone and Joint Surgery* published in 2003 [102]. This categorization was performed by an experienced orthopedic surgeon. 

### 3.5. Non-Diagnostic Studies 

Studies that were not classified as diagnostic looked at the use of computers in US imaging for tracking, segmentation, and measurement of cross-section area and echo texture. The two tracking studies looking at tendon and cartilage found excellent tracking results with AI [107,108]. One study even reported knee cartilage tracking results comparable to those of experienced surgeons [108]. Bone was primarily researched regarding segmenting as seen in three studies which reported automatic bone segmentation was accurate and comparable to existing techniques [109,110,111]. Seven studies investigated muscle for the purposes of measurement and segmentation [112,113,114,115,116,117,118]. Two studies looked at the gastrocnemius and reported they were able to automatically label ultrasound images and estimate neural output, length, and tension [115,116]. One study found it was possible to segment and track muscle on ultrasound images in real time, suggesting a potential usage for diagnosis [112]. 

### 3.6. Diagnostic Studies 

Twenty-two “diagnostic studies” could be further divided into screening, diagnosis, and prediction of prognosis, depending upon the role of AI/DL/CNN-based ultrasound in a clinical setting (Table 2). 

#### 3.6.1. Screening

Ultrasound is inherently an ideal imaging modality for screening due to its portability, cost-effectiveness, safety, and accessibility. AI can enhance the value of ultrasound as a screening tool by improving its diagnostic accuracy. 

Screening infants’ hips for hip dysplasia was the most studied use, with five diagnostic studies focusing on it. The results of these studies demonstrated that computer algorithms could successfully differentiate between diseased and healthy hips at a rate comparable to that of medical experts and the conventional Graf method [120,123,130,131,132]. 

Osteochondritis dissecans (OCD) of the humeral capitellum was another well-studied pathology, featuring in three diagnostic studies [135,138,139]. These studies indicated that DL-assisted ultrasound has a high accuracy for identifying and classifying OCD lesions. These studies’ results highlighted the potential use of DL-based ultrasound in screening baseball players for OCD. 

Another screening-based diagnostic study focused on osteoporosis and found a multichannel CNN-based ultrasound may be more accurate than a conventional quantitative ultrasound [124]. 

#### 3.6.2. Diagnosis

Carpal tunnel syndrome (CTS) was a frequent subject of investigation, with three studies, including one systematic review [119,121,126,133,134]. Two of these studies showed that the diagnosis of CTS could be performed with greater accuracy than that of radiologists [119,121]. 

Similarly, three studies investigating tendinopathy diagnosis with computer-guided ultrasound found that AI was able to detect Achilles, lateral elbow, and supraspinatus calcific tendinopathy with high diagnostic accuracy [122,129,136]. 

#### 3.6.3. Prediction

Two studies focused on the prediction of prognosis. In prognosis studies the machine learning software is trained on data (including clinical data, ultrasound images, laboratory data, etc.) to identify patterns and risk factors that may indicate the risk of developing disease.

One study found that DL was effective at predicting total knee replacement in patients with knee osteoarthritis [125]. Another study showed that machine learning was effective at predicting rheumatoid arthritis relapse [127]. 

### 3.7. Benefits of Utilizing AI/DL in Ultrasound Evaluation

Ultrasound is inherently operator-dependent, which can lead to variability in diagnostic accuracy. However, as demonstrated in recent diagnostic studies above, the integration of AI/DL into ultrasound evaluation significantly enhances the screening, diagnosis, and prediction of various MSK pathologies. These advancements underscore AI’s potential to transform ultrasound imaging into a more precise, reliable, and predictive modality in medical practice.

### 3.8. Limitations of AI/DL-Assisted Ultrasound 

#### 3.8.1. Image Quality Dependency 

AI algorithms are highly sensitive to image quality. As observed in the hip dysplasia studies, low-quality images could significantly impact the accuracy of AI interpretations [130,132]. The variability in ultrasound image acquisition techniques and equipment across different clinical settings posed a challenge for developing robust AI models. Consistent, high-quality ultrasound images across different operators and machines remain a hurdle that needs to be overcome. 

#### 3.8.2. Region of Interest (ROI) Sensitivity 

The accuracy of AI algorithms can be affected by variations in the selected region of interest. In osteochondritis dissecans of the humeral capitellum and carpal tunnel syndrome, adjusting the ROI improved consistency [133,138]. Optimal ROI selection across different pathologies and anatomical structures is crucial for reliable results. 

## 4. Utility of Artificial Intelligence in Ultrasound-Guided Surgery 

### 4.1. Literature Search 

Given the relatively novel and specialized nature of this section, initial structured database searches yielded limited relevant results. Therefore, we employed a snowball sampling approach to identify literature. This method involved identifying key papers in the field and systematically exploring their references (backward snowballing) and citations (forward snowballing). This approach allowed for the discovery of highly specific and relevant studies that might have been missed through conventional search strategies. While this method uncovered valuable research, it is important to note that it may not capture the entire breadth of available literature. 

### 4.2. AI in Ultrasound-Guided Surgery 

Our comprehensive literature review revealed no studies meeting the criteria for fully AI-integrated ultrasound-guided surgery as defined in our methodology. Currently, the field appears to be taking a staged approach, focusing on AI-enhanced assistive technologies rather than fully autonomous systems. 

### 4.3. Applications in Spine Surgery 

In spine surgery, AI-augmented ultrasound guidance has shown remarkable potential. Baka et al. developed an AI-based method to identify vertebral levels using ultrasound imaging. The method achieved 92–95% accuracy in correctly identifying vertebral levels in a test set of 19 patients, significantly outperforming traditional manual palpation techniques [140]. By combining pre-operative X-rays with intraoperative ultrasound, their method could offer a promising alternative to C-arm imaging, potentially reducing radiation exposure and improving workflow in operating rooms. 

### 4.4. Current State and Future Direction 

**Figure 4:**
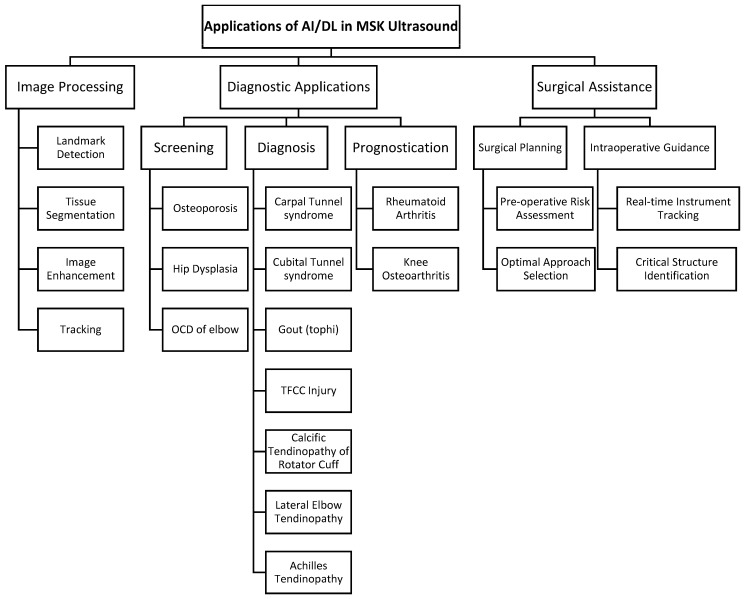
Summary of AI/DL applications in musculoskeletal ultrasound. AI/DL, artificial intelligence/deep learning; MSK, musculoskeletal; OCD, osteochondritis dissecans; TFCC, triangular fibrocartilage complex.

Real-time incorporation of AI while performing ultrasound-guided surgery is currently limited, mostly due to the nascency of both components. However, this does not preclude the use of AI techniques with ultrasound for the improvement of the perioperative experience. As the history of ultrasound evolved from diagnostic applications to interventional uses, the integration of AI in ultrasound technology is expected to transition from its current use in screening, diagnosis, and prediction to broader utilization in intervention and surgery (Figure 4).

## 5. Conclusions

Recent studies on ultrasound-guided surgery, particularly for soft tissue pathologies, have demonstrated a strong safety profile and efficacy comparable to traditional methods, with additional benefits such as pain reduction and quicker functional recovery. Despite ultrasound’s inherent operator dependency, which can lead to variability in diagnostic and therapeutic accuracy, our review demonstrated that integrating AI and deep learning into ultrasound imaging significantly improved the screening, diagnosis, and prediction of various musculoskeletal pathologies. These advancements underscore the potential of AI and deep learning to transform ultrasound, especially in ultrasound-guided procedures, into a more precise and reliable tool in musculoskeletal medicine. Further development of specialized devices for ultrasound-guided surgery, such as Tenex^®^, can further enhance the effectiveness of ultrasound as a tool for surgical guidance.

## Figures and Tables

**Table 1:** Clinical Studies of ultrasound-guided surgery above Evidence Level 3.

Primary Author	Year	Sample	Design	Pathology	Target Tissue	Key Findings
Wahezi [54]	2023	26 vs. 13	RCT	Adhesive capsulitis	Shoulder capsule	Ultrasound-guided PCHLR significantly improved post-procedure ROM compared to control.
Nikolaou [64]	2017	16 vs. 16	RCT	Trigger finger	Tendon	US-guided release resulted in shortened days off work, better cosmetic results, and no major complications compared to open surgery.
De la Fuente [46]	2021	47 vs. 42	RCT	CTS	Nerve	Hand functionality improved for ultrasound group; pain decreased significantly at 3-month follow-up.
Rojo-Manaute [45]	2016	46 vs. 46	RCT	CTS	Nerve	US guidance resulted in earlier functional recovery and significantly better pain scores without complications.
Kirschner [84]	2021	19 vs. 21	RCT	Chronic tendinopathy	Tendon	Pain scores in the PNT group were significantly lower than those in the PNT + LR-PRP group at 6 weeks, but no significant outcome differences.
Altahawi [103]	2020	23 vs. 10	RCT	CET tendinopathy	Tendon	Percutaneous tenotomy had similar outcomes to traditional surgery.
Alfredson [104]	2011	19 vs. 18	RCT	Achilles tendinopathy	Tendon	US-guided scraping shows good results with minimal complications, no significant difference between percutaneous and mini-open scraping.
Shomal [105]	2023	35	Meta-analysis	Tendinopathy fasciopathy	Tendon	PUNT (including dry needling) alleviated pain, improved function, and has low rate of complications and failures.
Boden [93]	2019	30 vs. 32	Comparative	Lateral and medial epicondylitis	Tendon	Both Tenex^®^ and PRP were successful in improving pain, function, and quality of life, but no significant difference between treatments.
Jacobson [106]	2016	15 vs. 15	Comparative	Gluteal tendinopathy	Tendon	US-guided tendon fenestration and PRP injection are effective for treatment of gluteal tendinosis with no significant difference between treatments.
Turner [92]	2024	17 vs. 13	Comparative	Plantar fascia	Tendon	PUT and PRP significantly decreased pain levels compared to only PUT.
Capa-Grasa [44]	2014	20 vs. 20	Comparative	CTS	Nerve	Ultra-MIS resulted in better recovery of functionality and symptoms in less post-operative time than mini-OCTR.
Hattori [97]	2020	11 vs. 15	Comparative	Chronic ankle instability	Ligament	US-guided anchor placement is accurate and anatomic for ATFL repair.
Wang [31]	2019	10 vs. 12	Comparative	Haglund deformity	Bone bursa	US-guided group had less pain and better function at 2 months compared to open surgery group.
Paczesny [10]	2021	20 vs. 15	Comparative	AT rupture	Tendon	US performed intraoperatively can minimize risk of sural nerve injury during percutaneous AT repair.
Lee [99]	2020	12 vs. 18	Comparative	AT rupture	Tendon	Percutaneous repair provides similar clinical outcomes, greater overall and aesthetic satisfaction levels, and minimal complications compared to open repair surgeries.

RCT, randomized controlled trial; CTS, carpal tunnel syndrome; PCHLR, percutaneous coracohumeral ligament release; ROM, range of motion; CET, common extensor tendons; US, ultrasound; PNT, percutaneous needle tenotomy; LR-PRP, leukocyte-rich platelet-rich plasma; PUNT, percutaneous ultrasound-guided needle tenotomy; PUT, percutaneous ultrasonic tenotomy; Ultra-MIS, ultra-minimally invasive surgery; mini-OCTR, mini-open carpal tunnel release; ATFL, anterior talofibular ligament; AT, Achilles tendon.

**Table 2:** Diagnostic studies utilizing AI/DL in musculoskeletal ultrasound imaging.

Primary Author	Year	Pathology	Evidence Level	Reference Standard	Key Findings
Faeghi [119]	2021	CTS	2	Nerve conduction test	Computer system performed better than two radiologists.
Lee [120]	2021	Hip dysplasia	4	Medical experts	DL model tested performed similarly to medical experts in dysplasia detection.
Chiu [121]	2022	RC (calcific tendinopathy)	2	Two experts	DL algorithm was accurate for the diagnosis of supraspinatus calcific tendinopathy.
Droppelmann [122]	2022	LET	4	Diagnosis by specialists	AI (specifically random forest model) detected LET with high diagnostic accuracy.
He [123]	2022	Hip dysplasia	4	Graf method	AI and 3D US-based automatic evaluation technology showed good agreement with the Graf method.
Luo [124]	2022	Osteoporosis	2	DEXA	Multichannel CNN could be more accurate than the conventional speed of sound model using quantitative US.
Matsuo [125]	2022	RA	2	Relapse	Excellent performance of ML at predicting RA relapse.
Shinohara [126]	2022	CTS	2	EPS	DL could detect carpal tunnel syndrome with high precision and accuracy.
Shinohara [127]	2022	Cubital tunnel syndrome	4	Electromyography	DL provided accurate prediction of cubital tunnel syndrome.
Shinohara [128]	2022	TFCC injury	4	MRI, CT arthrogram, and arthroscopy	DL detected TFCC with high accuracy comparable to MRI and CT arthrogram.
Tiulpin [129]	2022	Knee OA	2	TKR	DL-guided US was effective at predicting TKR.
Atalar [130]	2023	Hip dysplasia	4	Graf method	Successful differentiation of diseased and healthy hips.
Jaremko [131]	2023	Hip dysplasia	2	Orthopedic surgeons	All infants identified by AI-supported portable US were treated for hip dysplasia with 100% specificity.
Kinugasa [132]	2023	Hip dysplasia	4	Graf method	US with DL could assess hip dysplasia with high accuracy.
Lin [128]	2023	Gout (tophi)	4	Rheumatologist	It was possible to re-train deep CNN to identify the patterns of tophi in US images with accuracy.
Lyu [133]	2023	CTS	4	Not specified	Model performed best when median nerve epineurium was included in ROI.
Wu [134]	2023	CTS	3	Not applicable (systematic review)	In contrast to assessments by radiologists, US radiomics exhibited superior diagnostic performance in detecting CTS.
Shinohara [135]	2023	OCD of elbow	2	Radiographs and MRI	DL on US images identified OCD with high accuracy.
Wang [136]	2023	Achilles tendinopathy	2	Clinical diagnosis	US image-based radiomics achieved high diagnostic performance.
Yu [137]	2023	Scapulohumeral periarthritis	4	Diagnostic criteria	US combined with AI algorithm for scapulohumeral periarthritis is a simple method with high diagnostic efficiency.
Sasaki [138]	2024	OCD of elbow	4	Radiographs and orthopedic surgeon	Using DL with ROI focused on the humeral capitellum was effective at classification of OCD.
Takatsuji [139]	2024	OCD of elbow	2	Radiographs and orthopedic surgeon	Computer-assisted diagnosis system with DL achieved high accuracy using US images.

CTS, carpal tunnel syndrome; OA, osteoarthritis; OCD, osteochondritis dissecans; RA, rheumatoid arthritis; RC, rotator cuff; TFCC, triangular fibrocartilage complex; LET, lateral elbow tendinopathy; TKR, total knee replacement; AI, artificial intelligence; CNN, convolutional neural network; DL, deep learning; ML, machine learning: DEXA, dual-energy X-ray absorptiometry; EPS, electrophysiological study; CT, computed tomography; MRI, magnetic resonance imaging; US, ultrasound; ROI, region of interest.
